# Ubiquitin-specific peptidase 22 controls integrin-dependent cancer cell stemness and metastasis

**DOI:** 10.21203/rs.3.rs-2922367/v1

**Published:** 2023-06-16

**Authors:** Kun Liu, Qiong Gao, Yuzhi Jia, Juncheng Wei, Shuvam Chaudhuri, Shengnan Wang, Amy Tang, Nikita Mani, Radhika Iyer, Yang Cheng, Beixue Gao, Weiyuan Lu, Zhaolin Sun, Huiping Liu, Deyu Fang

**Affiliations:** Northwestern University; Northwestern university; Northwestern university; Northwestern university; Northwestern university; Northwestern university; Northwestern University; Northwestern University; Northwestern University; Northwestern University; Northwestern University; Northwestern University; Dalian Medical University; Northwestern University; Northwestern University

**Keywords:** Cancer stem cells, USP22, Integrins, metastasis

## Abstract

Integrins plays critical roles in connecting the extracellular matrix and actin skeleton for cell adhesion, migration, signal transduction, and gene transcription, which upregulation is involved in cancer stemness and metastasis. However, the molecular mechanisms underlying how integrins are upregulated in cancer stem cells (CSCs) remain as a biomedical mystery. Herein, we show that the death from cancer signature gene USP22 is essential to maintain the stemness of breast cancer cells through promoting the transcription of a group of integrin family members in particular integrin β1 *(ITGB1).* Both genetic and pharmacological USP22 inhibition largely impaired breast cancer stem cell self-renewal and prevented their metastasis. Integrin β1 reconstitution partially rescued USP22-null breast cancer stemness and their metastasis. At the molecular level, USP22 functions as a bona fide deubiquitinase to protect the proteasomal degradation of the forkhead box M1 (FoxM1), a transcription factor for tumoral *ITGB1* gene transcription. Importantly unbiased analysis of the TCGA database revealed a strong positive correlation between the death from cancer signature gene ubiquitin-specific peptidase 22 (USP22) and *ITGB1,* both of which are critical for cancer stemness, in more than 90% of human cancer types, implying that USP22 functions as a key factor to maintain stemness for a broad spectrum of human cancer types possibly through regulating *ITGB1.* To support this notion, immunohistochemistry staining detected a positive correlation among USP22, FoxM1 and integrin β1 in human breast cancers. Collectively, our study identifies the USP22-FoxM1-integrin β1 signaling axis critical for cancer stemness and offers a potential target for antitumor therapy.

## Introduction

Despite recent therapeutic advances in tumor treatment, metastasis to the nearby or distal organs remains the main cause of cancer-related death^1^. It has been proposed that only a small portion of primary tumor cells, termed cancer stem cells (CSCs), are responsible for metastasis ^2^. CSCs are a small population in tumor that are self-renewable, preferentially aggressive and responsible for cancer initiation, metastasis, and recurrence ^3^. Breast cancer stem cells (BCSCs), for example, have antioxidative, tumor sphere formation, and chemoresistance properties. Based on cell surface marker expression, BCSCs are CD44(+)/CD24(−/low) tumorigenic cells that initiate tumors in xenografts^4^. CD44 is a cell surface glycoprotein and stemness marker in BCSCs. CD44 binds to hyaluronic acid and mediates the interactions between cell/cell and cell/matrix proteins such as matrix metalloprotease and osteopontin ^5^. We have recently discovered that CD44 homophilic interactions and subsequent CD44-PAK2 interactions mediate tumor cluster aggregation and metastasis ^6^. While some progress has been made in characterization of CSCs over the last decade, the cellular and molecular mechanisms underlying how CSCs are generated and how their self-renewal is maintained remain largely unknown.

The ubiquitin-specific peptidase 22 (USP22) was initially identified as one of the 11 genes in cancer-related death signatures and referred to as the Polycomb/cancer stem cell signature group ^7^. Further survey of gene expression has shown that the elevated expression of USP22 correlates with poor prognosis in a variety of human tumors including the invasive breast cancer ^8^, ^9^. At the molecular level, we and others have recently demonstrated that USP22 functions as an oncogene by inhibiting cell apoptosis and promoting cell cycle progression through targeting cyclins, c-MYC, BMI-1, TRF1, and SIRT1, which controls p53 expression ^10, 11, 12, 13, 14, 15^. USP22 promotes chemotherapeutic resistance by inhibiting Bax-mediated apoptosis, improving HSP90 function, inhibiting Estrogen receptor a degradation, and driving EGFR recirculation ^16^. Genetic USP22 suppression inhibits cancer cell growth and induces apoptosis ^10, 13^. USP22 has been speculated to act as a critical cancer stem cell gene ^17^, however, the molecular pathways underlying if and how USP22 maintain cancer cell stemness and control CSC self-renewal remain to be fully defined.

In this study, we present evidence that USP22 is highly expressed in breast cancer stem cells and required for both breast cancer initiation and metastasis. Both genetic and pharmacological USP22 inhibition largely reduced the breast cancer stem cell pool through down-regulating integrin b1, also known as CD29, a cell surface glycoprotein that is critical in almost every step of cancer progression, including cancer initiation, proliferation, local invasion, and metastatic colonization of the new tissue ^18, 19^. Interestingly, integrin b1 has been used as a biomarker for isolating breast cancer stem cells ^20^. Indeed, reconstitution of integrin b1 expression fully rescued the BCSCs pool impaired by USP22 deficiency. At the molecular level, we identified the *ITGB1* transcription factor FoxM1 as a *de novo* substrate of the USP22 deubiquitinase. Therefore, USP22 controls breast cancer stem cell self-renewal through protecting FoxM1 from ubiquitination-mediated proteasomal degradation to enhance *ITGB1* transcription. Our study defines the USP22-FoxM-integrin b1 axis as a previously unappreciated pathway in breast cancer initiation and metastasis, that can be therapeutically targeted to antagonize invasive breast cancers.

## Results

### USP22 is required for the tumorigenicity of breast cancer stem cells.

**Figure 1:**
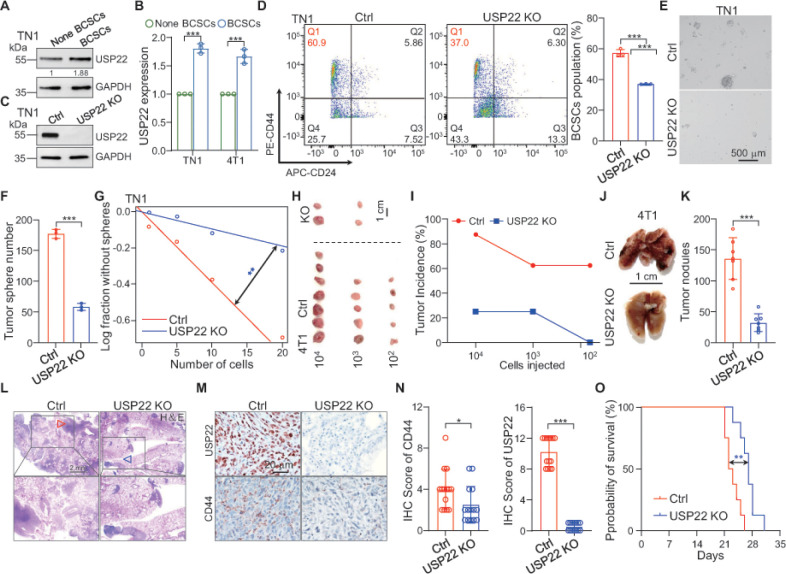
USP22 is required for BCSCs tumorigenicity. **A**. Immunoblot assessment of USP22 in the matched pairs of tumors sphere-enriched BCSCs and non BCSCs of TN1 cells. Band intensities of USP22 was quantified and the results are expressed as USP22/GAPDH levels relative to control cells. **B**. Quantification showing that USP22 was highly expressed in BCSCs than in none BCSCs. **C.** TN1 cells were transduced with single guide RNA (sgRNA) targeting USP22 or a scrambled control sgRNA, and knockout efficiency of USP22 was determined by immunoblot analyses. **D**. USP22 ablation decreases BCSCs (CD44^+^CD24^−^) population in TN1 cells determined by flow cytometry. Representative FACS data are shown. Quantification data showing that BCSCs population in USP22-deficent cells was dramatically decreased than control cells. **E**. Tumor sphere formation ability was evaluated in TN1 cells expressing either control or USP22 sgRNA, and the representative images of each group are shown. Scale bar, 500 mm. **F**. Silencing USP22 markedly impairs TN1 cells tumor sphere formation ability. **G**. The frequencies of tumor sphere formation of TN1 USP22 ablation or control cells determined by *in vitro* extremely dilution analysis. The significance of the difference between the indicated groups was evaluated by c^2^ test. n=10. **H**. Images of tumors developed from mice orthotopically implanted with indicated different gradients 4T1 USP22 ablation or control cells. n=8. **I**. Quantification showing that USP22 ablation impairs breast cancer initiation. **J**. Representative images of lung from mice intravenously injected with 4T1 cells expressed either control or USP22 sgRNA. Scale bar, 1 cm. **K**. The mice were humanely killed after 20 days injection of indicated 4T1 cells. The numbers of metastatic nodules in the lung were significantly decreased in mice injected with 4T1 USP22 knockout cells compared with the numbers in those injected with 4T1 control cells. **L**. The haematoxylin and eosin (H&E) staining show metastatic tumor. Scale bar, 2 mm. **M.** Immunohistochemical staining using anti-USP22 or CD44 antibodies were performed on metastatic nodules. Representative images of each group are shown. Scale bar, 20 mm. **N**. Quantification showing that USP22 knockout induced the decrease of CD44 positive cells. **O**. Kaplan-Meier survival curve of mice intravenously injected 4T1 cells expressed with or without USP22 sgRNA. Quantifications showing that injecting USP22 ablation cells extends mice survival relative to inject control cells. The significance of the difference between the indicated groups was evaluated by log rank test. n=8. The error bars show the mean ± SD. The significances of differences between different groups were determined by two-tailed Student’s t test. *, *** indicates *P* < 0.05, *P* < 0.001, respectively.

USP22 has been suggested as a cancer stem cell gene or death-from cancer signature gene and its high expression often predicts the poor clinical outcomes of cancer patients ^7^, but its role in maintaining CSC stemness remains to be defined. We sorted CD24^−^CD44^+^ breast CSCs from the patient-derived Luc2-eGFP (L2G)-labeled breast triple-negative (TN1) cancer cells ^6^ as well as in the murine breast cancer 4T1 cells (Figure s1 A), and found a significantly higher USP22 expression in breast CSCs compared to that in the CD24^+^CD44^−^ non-CSCs by western blotting (Fig. 1A & 1B and Figure s1 B). To decipher the USP22 functions in generating and/or maintaining breast cancer cell stemness, we generated USP22 targeted deletion in mouse 4T1 and human breast cancer-derived L2G^+^ TN1 cells by a CRISPR-Cas9 approach. A complete USP22 deletion was validated by immunoblot analysis (Fig. 1C & s1 C). Importantly, silencing USP22 dramatically reduced the CD24^−^CD44^+^ breast CSCs population in L2G^+^ TN1 and 4T1 cells (Fig. 1D and s1D & s1 E), indicating that USP22 is important for breast CSCs self-renewal. We then utilized a well-established tumor sphere formation assay ^21,22^ to evaluate the role of USP22 in breast CSCs self-renewal. Indeed, the tumor sphere formation from both patient derived L2G^+^ TN1 and mouse 4T1 breast cancer cells was largely impaired by USP22 CRISPR deletion, which was further confirmed by an *in vitro* extremely limiting dilution assay (Fig. 1E–G, s1F-H). Consistently, USP22 inhibition in 4T1 cells resulted in a substantial reduction in colony formation (Figure s1I & J). Therefore, these results indicate that USP22 is required to maintain an optimal CSC population, possibly by controlling CSC self-renewal *in vitro.*

CSCs are a critical small population of cancer cells with potent capability for tumor initiation. To test USP22’s function in promoting tumor initiation *in vivo.* We orthotopically injected 10^2^, 10^3^, and 10^4^ USP22 knockout or control 4T1 breast cancer cells into BALB/c mice. Surprisingly, in contrast to the fact that five out of eight mice implanted with 10^2^ WT 4T1 cells developed cancer three months after implantation, none of the eight mice received with USP22-deficient 4T1 cells developed breast cancer. Even when a higher number of 4T1 cells, 10^3^ and 10^4^, were orthotopically injected, USP22 deletion dramatically inhibited the development of syngeneic tumors (Fig. 1H & 1I), indicating that USP22 is critical for *in vivo* tumor initiation. Cancer metastases, per prevailing theory, are predominantly initiated by rare cancer cells that bear stem cell properties ^23, 24^. We then determined whether USP22 exerted a driving role in breast cancer metastasis by intravenously injection of 4T1 USP22-null or its control WT cells into BALB/c mice. As expected, USP22 deletion dramatically inhibited 4T1 cancer colonization to the lung by reducing more than 60% of tumor nodules with further reduced metastatic foci size (Fig. 1J–L). Immunohistochemistry staining confirmed the deletion of USP22 and detected a significant decrease in the levels of stem cell marker CD44 expression in lung metastasis (Fig. 1M & 1N). As a consequence, USP22 ablation significantly improved the overall survival of the mice with 4T1 lung metastasis (Fig. 10). Collectively, our results revealed that USP22 play an important role in breast CSC maintenance, which is critical for breast cancer initiation and metastasis.

### USP22 promotes breast CSC self-renewal through upregulating ITGB1 expression

**Figure 2:**
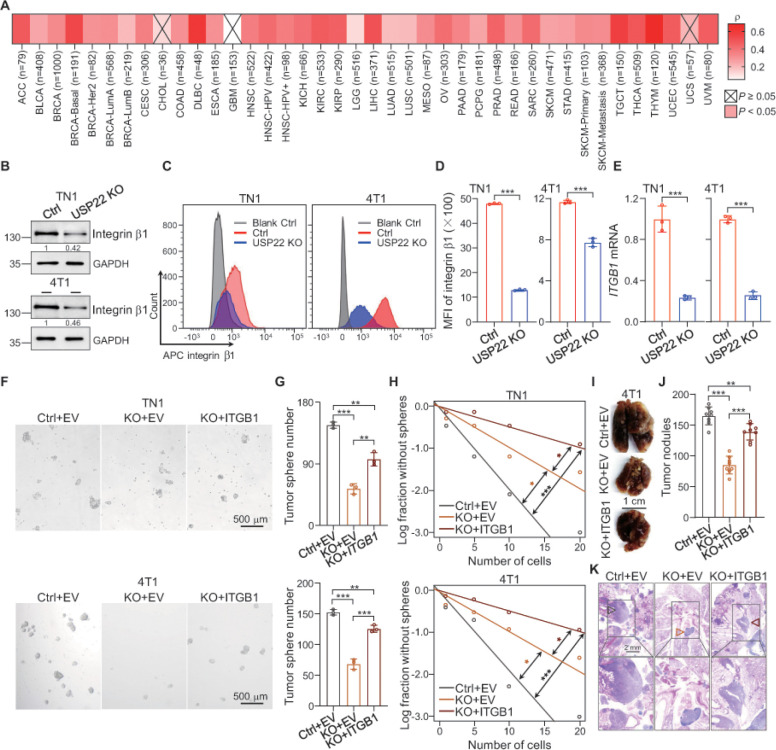
Depleting USP22 prohibits integrin b1 expression. **A.** Heatmap of correlation between *USP22* and *ITGB1* expression in various cancer types. **B**. Indicated protein expression in TN1 and 4T1 cells expressing either control or USP22 sgRNA were determined by immunoblot analysis. Band intensities of integrin b1 was quantified and the results are expressed as integrin bVGAPDH levels relative to control cells. **C**. Integrin b1 level was decreased in USP22 ablation cells determined by flow cytometry. Representative FACS data are shown. **D**. Mean fluorescence intensity (MFI) of integrin b1 level in TN1 and 4T1 USP22-deficent or control cells was quantified. **E.** The mRNA expression of *ITGB1* (gene encoding for integrin b1) in TN1 and 4T1 USP22-deficent or control cells was determined by real-time PCR. b-actin was used as internal control. **F-G** The representative images (F) and number (G) of tumor sphere formed from TN1 and 4T1 cells transduced with integrin b1 in the setting of USP22 depletion. Scale bar, 500 mm. **H**. The frequencies of tumor sphere formation of indicated cells. Quantifications showing that introduction of integrin b1 restores tumor sphere formation frequency caused by USP22 depletion evaluated by *in vitro* extremely limiting dilution analysis. The significance of the difference between the indicated groups was evaluated by c^2^ test. n=10. **I**. Representative images of lung from mice intravenously injected with indicated cells. Scale bar, 1 cm. **J**. The numbers of metastatic nodules in the lung from mice intravenously injected with indicated cells. **K**. H&E staining of lung metastasis of indicated group. Scale bar, 2 mm. The error bars show the mean ± SD. The significances of differences between different group were determined by two-tailed Student’s t test. **, *** indicates *P* < 0.01, *P* < 0.001, respectively.

Integrin family members are known as key regulators in cancer cell stemness, epithelial-mesenchymal transformation and extracellular matrix to initiate the metastatic process for multiple cancer types including breast cancer ^19, 25^. Importantly, unbiased analysis of the public database TMIER2 ^26^ revealed a strong and statistically significant positive correlation nearly in all types, 37 out of total 40 of human cancers (Fig. 2A and Supplementary table 1). Further analysis of integrin a and b family revealed a positive correlation of several integrin family members in particular integrin a1,2, 8 & 9 (Figure s2A and Supplementary table 1). These results suggest a possibility that USP22 regulates cancer stem cell self-renewal through regulating the transcription of some of integrin family members. Indeed, comparison analysis by western blotting and flow cytometry of integrin family members between WT and USP22-null breast cancer cells detected a dramatic reduction in integrin b1 expression by USP22 inhibition in both mouse 4T1 and patient-derived L2G^+^ TN1 cells (Fig. 2B–D). USP22 appears to positively regulate integrin b1 expression at the transcriptional level because its targeted deletion resulted in a more than 70% reduction in *ITGB1* mRNA levels (Fig. 2E). In addition to integrin b1, USP22 deletion led to a modest but statistically significant reduction in several additional integrin family members including integrin a1–6 and integrin b2–3, b5–7, but not b4, b8 and a7–8 expression (Figure s2B), In contrast, integrin b6 expression is slightly increased in USP22-null breast cancer cells (Figure s2B). Therefore, USP22 appears to regulate the expression of multiple integrin family members with b1 as the dominant one.

We then focused on studying the functional consequences of USP22-mediated integrin b1 upregulation and assessed whether USP22 maintains breast CSC self-renewal and promotes breast cancer metastasis through integrin b1 upregulation by ectopic reconstitution of *ITGB1* in USP22 knockout cells (Figure s2C-E). Indeed, ectopic *ITGB1* expression partially rescued tumor sphere formation from in both mouse 4T1 and patient-derived L2G^+^ TN1 USP22-deficient breast cancer cells (Fig. 2F–H). Consequently, expression of integrin b1 largely, but not totally restored 4T1 breast cancer lung metastasis of USP22-null cells as documented by analyzing both lung tumor nodule numbers and the metastatic foci size (Fig. 2I–K). Collectively, these results demonstrate that USP22 enhances BCSCs tumorigenic potential, in part, through integrin b1 upregulation.

### USP22 functions as a de novo FoxM1-specific deubiquitinase in breast cancer cells.

**Figure 3:**
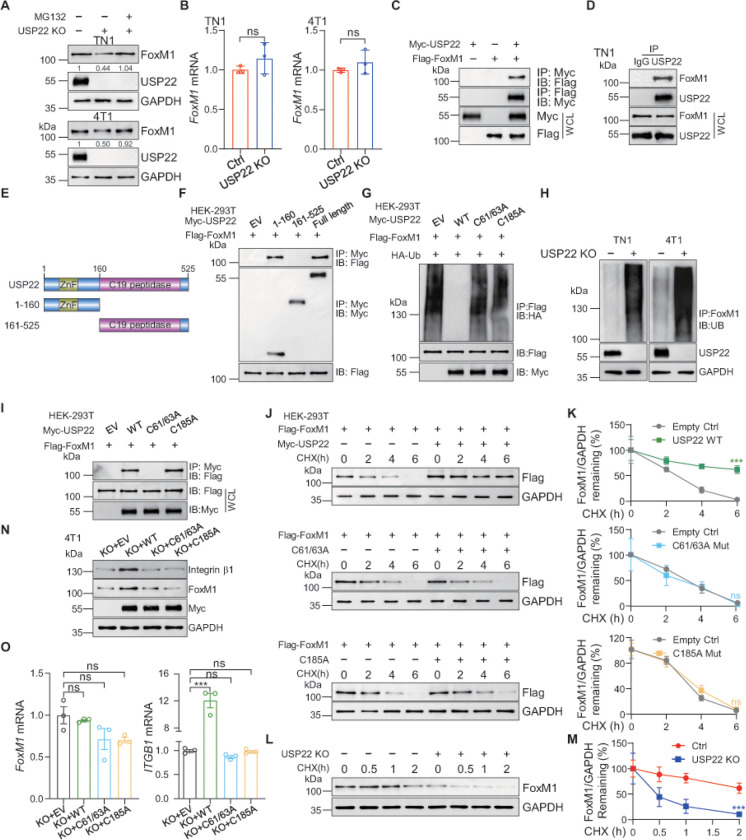
USP22 interacts with and stabilizes FoxM1. **A.** TN1 and 4T1 stably expressing control or USP22 sgRNA were treated with or without the proteasome inhibitor MG132 (10 mM, 12 h), and then FoxM1 protein expression level was evaluated. Band intensities of FoxM1 were quantified and the results are expressed as FoxM1/GAPDH levels relative to control cells. **B.** Total RNA was isolated from TN1 and 4T1 cells stably expressing control or USP22 sgRNA. The mRNA levels of *FoxM1* were determined by real-time PCR. b-actin was used as internal control. ns means no significant difference. **C**. Interaction of USP22 with FoxM1. HEK-293T cells were transiently transfected with Flag tagged FoxM1 and Myc tagged USP22. Cell extracts were immunoprecipitated (IP) using primary antibodies against Myc and then subjected to immunoblotting (IB) analysis. WCL means whole cell lysates. **D**. Endogenous USP22 and control IgG were immunoprecipitated from TN1 cell lysates and then subjected to immunoblotting for analyzing associated proteins. Rabbit IgG was used as the isotype control. **E.** Schematic representation of the N-terminal Myc-tagged full-length USP22, and various corresponding truncation mutants. **F**. HEK-293T cells were transfected with the indicated truncated constructs, followed by IP with Myc antibody and followed by immunoblot (IB) with antibodies against Flag. EV means empty vector. **G**. HEK-293T cells were transiently transfected with Flag tagged FoxM1, HA tagged ubiquitin and Myc tagged USP22 or USP22 C61/63A, C185A mutant. Cell extracts were IP using primary antibodies against Flag and then subjected to IB analysis to analyze FoxM1 ubiquitylation linkage. **H**. TN1 and 4T1 stably expressing control or USP22 sgRNA cell lysates were subjected to IP with FoxM1 antibody, followed by IB with antibodies against ubiquitin. Cells were treated with 10 mM MG132 for 12 hours before harvesting. **I**. HEK-293T cells were transiently transfected with Flag tagged FoxM1 and Myc tagged USP22 or USP22 C61/63A, C185A mutant. Cell extracts were IP using primary antibodies against Myc and then subjected to IB analysis. **J**. HEK-293T cells were co-transfected with Flag tagged FoxM1 and Myc tagged USP22 WT or USP22 C61/63A, C185A mutant for 24 h, followed by treating with 20 mg ml^−1^ cycloheximide for the indicated times, and cell lysates were subjected to immunoblot with indicated antibodies. CHX means cycloheximide. **K**. Quantification showing that overexpression USP22 WT, but not USP22 C61/63A, C185A mutant augments FoxM1 half-life. Quantification of FoxM1 relative to GAPDH was quantified by Image J. n=3. **L**. 4T1 USP22-deficent or control cells were treated with 20 mg ml^−1^ CHX for the indicated times and cell lysates were examined by immunoblotting. **M**. Quantification showing that USP22 ablation attenuates FoxM1 half-life. FoxM1 band intensity was quantified and the results are expressed as FoxM1/GAPDH levels relative to untreated cells. n=3. **N**. Immunoblot analyses of indicated proteins of 4T1 USP22-deficent cells transduced with USP22 WT or indicated mutants. Enforced expression of USP22 WT, but not indicated mutants, rescued the level of FoxM1 and integrin b1 in USP22-deficent cells. **O**. 4T1 USP22-deficent cells were transiently transduced with USP22 WT or indicated mutants for 48 h. The mRNA levels of *FoxM1* or *ITGB1* were determined by real-time PCR. Enforced expression of USP22 WT, but not indicated mutants, rescued the level of *ITGB1,* but not *FoxM1,* mRNA levels in USP22-deficent cells. b-actin was used as internal control. The error bars show the mean ± SD. The significances of differences between different group were determined by two-tailed Student’s t test. *** indicates *P* < 0.001.

The fact that USP22 deletion reduced *ITGB1* mRNA expression suggest that USP22 regulates integrin b1 expression at transcriptional level. Indeed, western blotting analysis revealed a significant reduction in the protein expression of FoxM1, a critical transcription factor for *ITGB1* expression ^27^, in USP22-null breast cancer cells (Fig. 3A). In contrast, USP22 ablation did not alter *FoxM1* mRNA levels (Fig. 3B). Together with the fact that USP22 is a deubiquitinase, these results imply that the USP22 exerts its regulatory function on FoxM1 protein expression at the post-transcriptional level. Indeed, treatment of USP22-null cells with the proteasome inhibitor MG132 largely restored FoxM1 expression to a level comparable to that in WT breast cancer cells (Fig. 3A). By contrast, treatment with NH_4_Cl, an inhibitor of endosome-lysosome degradation pathway, fails to protect FoxM1 from degradation (Figure s3A), suggesting that USP22 promotes FoxM1 level through inhibiting its proteasomal degradation.

As a deubiquitinase, USP22 exerts its biological function largely through protecting its downstream substrates from ubiquitination-mediated degradation ^28^. Accordingly, we speculated that USP22 could be a deubiquitinase of FoxM1. Indeed, USP22 interaction with FoxM1 was detected in HEK-293T cells transiently transfected Myc-USP22 and Flag-FoxM1, but not in control cells transfected with Flag-FoxM1 or Myc-USP22 alone (Fig. 3C). The endogenous interaction between USP22 and FoxM1 in patient-derived breast cancer L2G^+^ TN1 cells was further validated (Fig. 3D and s3B). USP22 protein carries an N-terminal zinc finger and C-terminal U19 peptidase catalytic domain (Fig. 3E). Truncated mutation analysis revealed that the zinc finger-containing N-terminus is sufficient for USP22 interaction with FoxM1, while the C-terminus ubiquitin-specific peptidase domain is not involved in mediating its FoxM1 interaction (Fig. 3F). These results indicate that FoxM1 physically interacts with USP22 in breast cancer cells.

A ubiquitin-specific peptidase often inhibits ubiquitination of its interacting proteins ^29^. Thus, we determined the effect of USP22 on FoxM1 ubiquitination. Higher molecular weight bands were detected in FoxM1 immunoprecipitants, indicating FoxM1 is ubiquitinated possibly by its endogenous E3 ubiquitin ligases such as FBWX7 ^30^. Importantly, transient USP22 expression largely diminished FoxM1 ubiquitination (Fig. 3G). Conversely, loss of USP22 expression resulted in a significant increase in FoxM1 ubiquitination in both mouse 4T1 and patient-derived breast cancer cells (Fig. 3H). Our data indicate physical interaction between USP22 and FoxM1 is required for USP22-mediated suppression of FoxM1 ubiquitination, because mutation of cystines 61 and 63, which disrupts the zinc finger structure and its interaction with FoxM1 (Fig. 3I), totally abolished USP22 activity in suppressing FoxM1 ubiquitination (Fig. 3G). As expected, expression of the catalytically inactive deubiquitinase, through C185A mutation of USP22, failed to inhibit FoxM1 ubiquitination despite not altering its interaction with FoxM1 (Fig. 3F & 3G). These results indicate that USP22 is a bona fide FoxM1-specific deubiquitinase in breast cancer cells. In concordance with this conclusion, USP22 overexpression dramatically prolonged FoxM1 half-life as measured by pulse-chase analysis (Fig. 3J & 3K). Consistent with the ubiquitination data, neither USP22 C185A nor C61/63A mutant sustained FoxM1 stability (Fig. 3J & 3K). In line with this, USP22 ablation dramatically decreased FoxM1 half-life (Fig. 3L & 3M). Consistently, re-expression of WT USP22, but not its mutants restored integrin b1 expression in USP22-null breast cancer cells (Fig. 3N & 3O). These results define USP22 as a *de novo* FoxM1 deubiquitinase in breast cancer cells to protect FoxM1 from ubiquitination-mediated proteasomal degradation for upregulating integrin b1 expression.

### USP22 promotes integrin b1 expression through FoxM1 stabilization.

**Figure 4:**
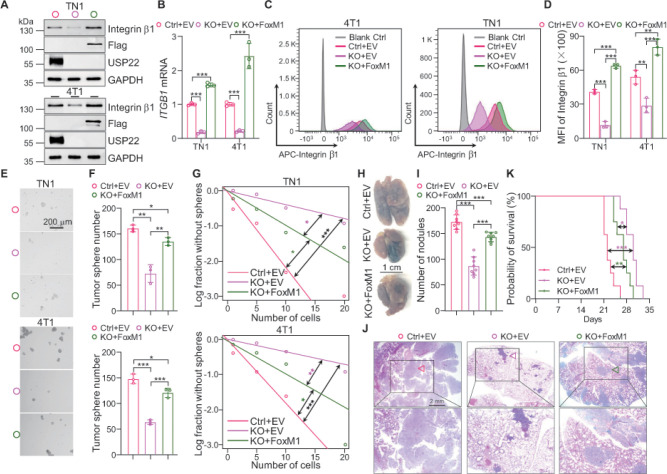
FoxM1 introduction partially rescues the suppressive effects caused by USP22 depletion. **A.**Immunoblot analysis of integrin b1, Flag and USP22 in TN1 and 4T1 cells transduced with Flag-FoxM1 in the setting of depleted USP22. Enforced expression of FoxM1 completely rescued the protein level of integrin b1 in USP22-deficent cells. **B**. The mRNA levels of *ITGB1* in TN1 and 4T1 cells were determined by real-time PCR. Enforced expression of FoxM1 rescued the mRNA level of *ITGB1* in USP22-deficent cells. b-actin was used as internal control**. C**. Ectopic expression of FoxM1 completely rescued the protein level of integrin b1 in USP22-deficent cells determined by flow cytometry. Representative FACS data are shown. **D**. Quantification showing that ectopic expression of FoxM1 completely rescued the protein level of integrin b1 in USP22-deficent cells. **E**. Tumor sphere formed from TN1 and 4T1 cells transduced with Flag-FoxM1 in the setting of depleted USP22. The representative images of tumor sphere are shown. Scale bar, 500 mm. **F.** Quantifications showing that FoxM1 expression partially rescues the decreased tumor sphere formation ability caused by USP22 depletion. **G**. The frequencies of tumor sphere formation in TN1 and 4T1 cells expressing Flag-FoxM1 or empty control in the setting of USP22 deficient. **H**. Representative images of lung derived from mice injected 4T1 cells expressing either control or USP22 sgRNA in combination with vector control or Flag-FoxM1. Scale bar 1 cm**. I.** Quantification result showing that introduction of Flag-FoxM1 partially rescues the inhibitory effects caused by USP22 depletion. **J**. H&E staining of lung metastasis of indicated group. Scale bar, 2 mm. **K**. Kaplan-Meier survival curves of mice implanted with indicated cells. Quantification showing that ectopic expression of FoxM1 in 4T1 USP22-deficient cells shorten mice survival than USP22-deficient cells. Significance testing was done by log rank test. The error bars show the mean ± SD. The significances of differences between different group were determined by two-tailed Student’s t test. *, **, *** indicates *P* < 0.05, *P* < 0.01, *P* < 0.001, respectively.

FoxM1 has been identified as an integrin β1 transcription factor thereby promoting breast cancer progression ^27^, implying a possibility that USP22 controls breast cancer cell *ITGB1* expression through FoxMl stabilization. Indeed, reconstitution of FoxM1 expression fully restored the endogenous integrin b1 expression in both USP22-null 4T1 and L2G^+^ TN1 breast cancer cells as determined by western blotting and qRT-PCR (Fig. 4A & 4B), which was further confirmed by flow cytometry (Fig. 4C & 4D). In contrast, we observed that FoxM1 expression fails to rescue integrin b2–7 expression (Figure s4A). These results support our hypothesis that USP22 specifically promote integrin b1 expression through FoxM1 stabilization. Consistent with this, we observed that ectopic expression of FoxM1 largely restored the tumor sphere formation ability of USP22-deficent breast cancer cells (Fig. 4E–G). Likewise, the impaired ability in colony formation of 4T1 breast cancer cells by USP22 depletion was largely rescued by exogenous FoxM1 expression (Figure s4B & s4C).

We also noticed that, while FoxM1 expression fully rescued integrin b1 expression both in USP22-null 4T1 and TN1 breast cancer cells, but their sphere and colony formation were only partially restored by FoxM1 re-expression (Fig. 4E–G). We then utilized the lung metastasis model to further illustrate the role of USP22-FoxM1-integrin b1 pathway in breast cancer tumorigenesis in BALB/c mice. Indeed, in contrast to the fact that USP22 deletion resulted in a more than 50% reduction in lung metastases 4T1 cancer nodules, FoxM1 re-introduction restored USP22-null 4T1 cancer lung metastasis to a level of about 85–90% of the WT (Fig. 4H–J). As a consequence, FoxM1 expression dramatically attenuated but not totally abolished the protection of mice from lung metastasis-induced lethality by USP22 targeted inhibition (Fig. 4K). Collectively, these results indicate that USP22 promotes breast cancer metastasis at least partially, through promoting FoxM1-mediated integrin b1 expression.

### Pharmacological inhibition of USP22 abrogates BCSCs tumorigenicity.

**Figure 5:**
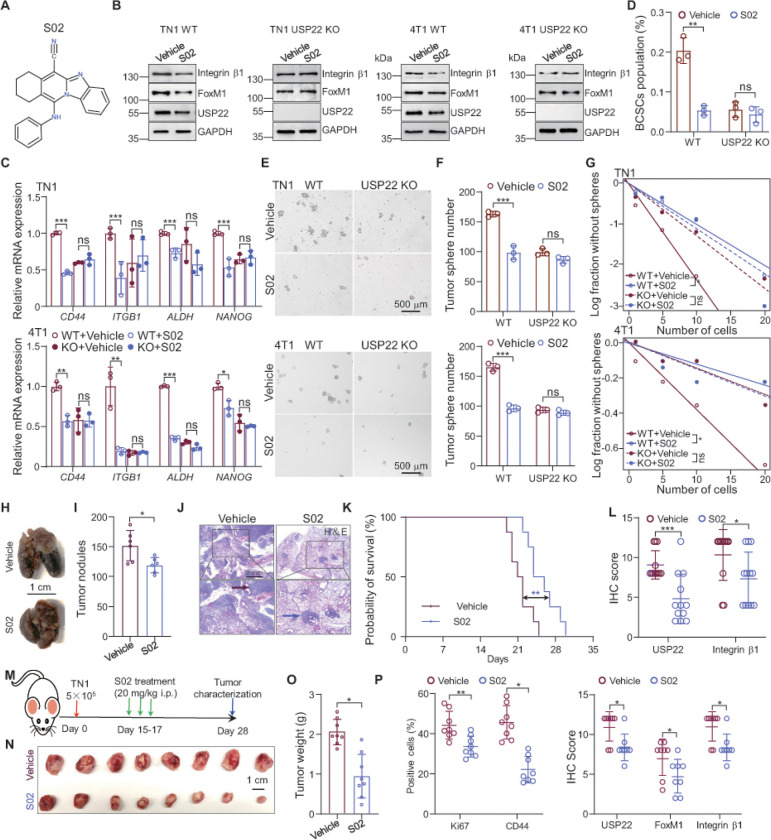
The USP22 inhibitor attenuates breast CSCs self-renewal. **A**. The chemical structure of USP22 inhibitor S02. **B**. TN1 and 4T1 WT or USP22-null cells were treated with 20 mM S02 for 24 h. Cell lysates were analysed by immunoblotting using the indicated antibodies. Dimethyl sulfoxide (DMSO) vehicle was used as a control. **C**. TN1 and 4T1 WT or USP22-null cells were treated with or without 20 mM S02 for 48 h. The mRNA levels of indicated genes in TN1 and 4T1 cells were determined by real-time PCR. b-actin was used as internal control. **D**. 4T1 WT or USP22-null cells were treated with 20 mM S02 for 48 h, the cells were subsequently stained with CD44 and CD24 antibodies, and then analyzed by flow cytometry. S02 treatment decreases BCSCs (CD44^+^CD24^−^) population determined by flow cytometry. Quantification data are shown. **E**. Tumor sphere formation ability was evaluated in TN1 and 4T1 cells treated with or without 20 mM S02 for 10 days. Representative images of each group are shown. Scale bars, 500 mm. **F**. Quantification showing that tumor sphere formation ability was restricted by S02 treatment in TN1 and 4T1 WT, but not USP22-deficient cells. **G**. *In vitro* extremely limiting dilution assay by plating gradient numbers of TN1 and 4T1 control or USP22 ablation cells showed the frequencies of tumor sphere formation in indicated cells treated with 20 mM S02 for 10 days. **H**. Representative mages of lungs from mice given intravenous injection of 5×10^4^ 4T1 cells. 24 hours later, mice were randomized into treatment groups and treated with S02 (10 mg/kg), or vehicle control by intraperitoneal injection six times (once every day). **I**. Tumor nodules on the lung of mice injected with S02 or vehicle control. Scale bar, 1 cm. **J**. The mice were humanely killed after 20 days injection of 4T1 cells. The H&E staining sections show representative metastatic tumour. Scale bar, 2 mm. **K**. 4T1 cells (5×10^4^ cells per mouse) were intravenously injected into BLAB/c mice. Mice were treated as described in H. The survival of mice was evaluated (n = 8. Kaplan-Meier plotter with two-sided log-rank test). **L**. Immunohistochemical staining of sections from nodules in the lung as in J stained with antibody against USP22 and integrin b1. **M**. The scheme for mouse breast cancer treatment model. TN1 cells (5×10^4^ cells per mouse) were orthotopically injected into NOD/SCG mice. Two weeks later when the tumors were reached to around 100 mm^3^, mice were randomized into treatment groups and treated with S02 (20 mg/kg) or vehicle control by intraperitoneal injection six times (twice a day). **N**. Images of xenograft tumors after orthotopically injecting TN1 cells and treated with vehicle or S02 by the indicated conditions. Scale bar, 1 cm. **O**. Weights of xenograft tumor treated with vehicle or S02. **P**. Immunohistochemical analysis of sections from xenograft tumors treated with vehicle or S02 stained with indicated antibodies. Three individual samples were analyzed and quantification data are shown. The error bars show the mean ± SD. The significances of differences between different group were determined by two-tailed Student’s t test. *, **, *** indicates *P* < 0.05, *P* < 0.01, *P* < 0.001, respectively.

Our discovery that genetic USP22 deletion hindered breast cancer stem cell self-renewal and inhibited their lung metastasis provides a rationale for USP22 targeting in anticancer therapy. We first analyzed the effects of pharmacological USP22 inhibition on BCSCs self-renewal using a small molecule inhibitor USP22i-S02 that we recently identified (Fig. 5A) ^31^. Similar to our observation from USP22 CRISPR KO studies, treatment of breast cancer cells 4T1 and TN1 significantly inhibited both integrin b1 and FoxM1 expression. Consistent with our previous observations, S02 treatment also reduced USP22 expression levels presumably because USP22 is a deubiquitinase of itself (Fig. 5B). Further addition of the proteasomal inhibitor MG132, but not with lysosome inhibitor NH_4_Cl, largely rescued FoxM1 protein levels from USP22i-S02 treatment (Figure s5A & s5B), confirming our observation that USP22 inhibition facilitates proteasomal FoxM1 protein degradation. In line with this, treatment of 4T1 cells with S02 dramatically shortened FoxM1 protein half-life (Figure s5C & 5D). As expected, S02 treatment suppressed *ITGB1* and other stemness related genes expression, including *CD44, ALDH,* and *NANOG* (Fig. 5C). In contrast, S02 treatment did not alter *FoxM1* mRNA transcription (Figure s5E). These results confirm that USP22 is a positive regulator for FoxM1-mediated *ITGB1* expression in breast cancer cells by an orthogonal pharmacological approach.

We next set out to determine the effects of USP22 pharmacological inhibition on breast cancer stem cell self-renewal. As expected, S02 treatment reduced breast CSCs population for more than 80%, to a level that is comparable to USP22 knockout (Fig. 5D and s5F). Importantly, treatment of USP22-null breast cancer cells did not further reduce the frequency of breast cancer stem cells (Fig. 5D and s5F), supporting the high specificity of this USP22-specific small molecule inhibitor. Consequently, treatment with S02 significantly impaired breast cancer cell sphere and colony formation capability (Fig. 5E & F and s5G & H). Further *in vitro* extremely limiting dilution assay confirmed that S02 inhibited breast CSCs self-renewal (Fig. 5G), implying for its great therapeutic potential in treatment breast cancer. We then used the preclinical 4T1 breast pulmonary metastasis model to illustrate the potential anti-metastatic effect of S02 (Figure s5L). Of note, a six-day treatment with S02 after tail vein injection of 4T1 breast cancer cells resulted in a significant reduction in 4T1 breast cancer lung metastasis and prolonged mice survival (Fig. 5H–K). Further immunohistochemistry analysis of the lung metastatic cancers detected a reduction in both integrin b1 and FoxM1 levels in the S02 treatment groups (Fig. 5L). Similar to our recent study, administration of S02 did not show any detectable toxicity because the mice body weight was unaltered (Figure s5J), and further hematoxylin-eosin (H&E) staining did not detect obvious liver damage in S02 treatment mice (Figure s5K). Therefore, these results indicate that pharmacological USP22 targeting is a safe and effective therapy in treatment of triple negative breast cancers.

We then further evaluated the therapeutic potential of USP22i-S02 in a patient-derive xenograft model by orthotopically implanting TN1 cells to immune compromised RAG1 mutant mice (Fig. 5M). Intriguingly, a 3-day treatment of pre-established PDX tumor significantly hindered patient derived xenograft tumor growth (Fig. 5N & 5O). Further characterization by IHC staining show that the levels of USP22, FoxM1 and integrin b1 protein expression by USP22i-S02 treatment, which consequently inhibited the breast cancer cell growth because the percentage of Ki-67^+^ proliferative cells was dramatically decreased. Importantly, we detected a significant reduction in CD44^+^ breast cancer cells in the S02 treat group, implying that USP22 pharmacological inhibition attenuates either the breast cancer stem cell self-renewal or their survival (Fig. 5P). Therefore, pharmacological inhibition of USP22 achieves represents a potentially efficacious treatment for breast cancer and metastasis.

### Positive correlation of USP22, FoxM1 and integrin b1 in human breast cancer.

**Figure 6:**
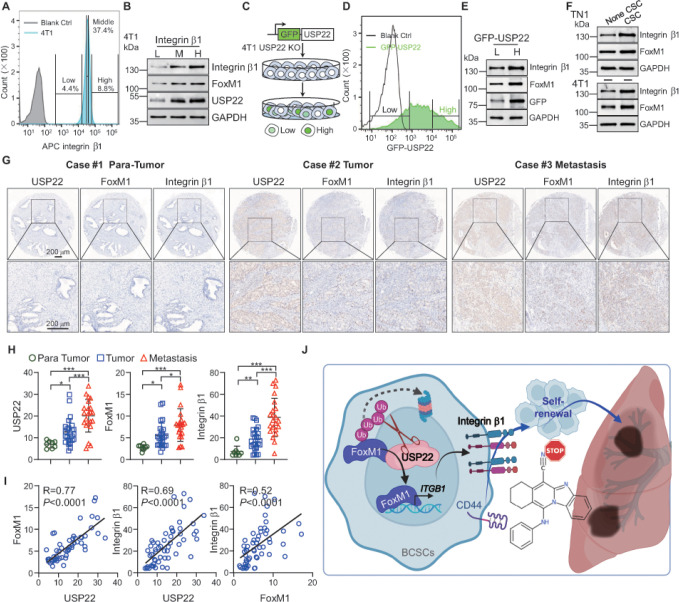
Clinical significance of USP22/FoxM1/Integrin b1 signaling axis in breast cancer. **A**. Flow cytometry was used to isolate low, middle and high integrin b1 cell from 4T1 cells. **B**. Immunoblot analysis for USP22, FoxM1, and integrin b1 in 4T1 cells isolated according to integrin b1 intensity. L, M, H indicate integrin b1 intensity low, middle, and high, respectively. **C**. The scheme for GFP-USP22 knock-in 4T1 USP22 knockout cells. **D.** FACS sorting of GFP^low^ or GFP^high^ cells isolated from GFP-USP22 knock-in in 4T1 USP22 knockout cells. Representative FACS data are shown. **E**. Immunoblot analysis for FoxM1 and integrin b1 of USP22 knock-in in 4T1 USP22 knockout cells isolated according to GFP intensity. **F**. BCSCs and none BCSCs were isolated from TN1 and 4T1 cells, respectively. The cell extracts were analyzed by immunoblotting using FoxM1 and integrin b1 antibody. **G.** Immunohistochemical staining of tissue microarray #1 including 55 specimens (n=8 breast para-tumor specimens, n=20 breast cancer specimens, and n=22 metastatic specimens) for USP22, FoxM1 or integrin b1. Representative consecutive sections from 3 specimens are shown. Scale bars: 200 mm. The clinicopathological characteristics of tissue microarrays are shown in Supplementary Table 2. **H**. The integrated optical density of USP22 (left panel), FoxM1 (middle panel) or integrin b1 (right panel) was compared with those in indicated groups. **I**. Linear regression analysis of the integrated optical density of USP22 and FoxM1 (left panel), USP22 and Integrin b1 (middle panel), and FoxM1 and Integrin b1 (right panel) showed a significant positive correlation. Pearson’s R correlation test and the Pearson correlation coefficients are shown in the matrix. n=55. **J**. Proposed working model. USP22 is a *de novo* FoxM1 deubiquitinase, which plays essential roles in triggering transcriptional activation of *ITGB1,* thereby promoting breast cancer stem cells self-renewal and drives breast cancer metastasis. The error bars show the mean ± SD. The significances of differences between different group were determined by two-tailed Student’s t test. *, **, *** indicates *P* < 0.05, *P* < 0.01, *P* < 0.001, respectively.

Our data collectively documented that USP22 maintain breast cancer stemness in part through stabilizing *ITGB1* transcription factor FoxM1 to promote breast cancer growth and metastasis, which define a previously unknown USP22-FoxM1*-ITGB1* pathway in breast cancer pathogenesis. Further analysis of the sorted integrin b1^low^, integrin b1^middle^ and integrin b1^high^ 4T1 cells revealed a gradual elevation in USP22 and FoxM1 expressions (Fig. 6A & 6B). We then generated a green fluorescent protein (GFP)-USP22 fusion knock-in in 4T1 cells with the endogenous USP22 ablation (Fig. 6C). Consistently, the expression of both FoxM1 and *ITGB1* are profoundly increased in USP22^high^ comparing to that in USP22^low^ 4T1 knock-in cells (Fig. 6D & E). Further, a significant increase in integrin b1 and FoxM1 in breast CSCs versus none breast CSCs population was observed (Fig. 6F).

To further determine the critical roles of the USP22-FoxM1-integrin b1 in breast cancer pathogenesis, we utilize the immunohistochemistry staining determined the expression of USP22, FoxM1, and integrin b1 protein in human breast cancer tissue microarray (Supplementary table 2). As expected, the protein levels of USP22, FoxM1, and integrin b1 was markedly higher in the breast tumor tissues than those in begin tumors (Fig. 6G, 6H & s6A-D), and levels were even further elevated in metastatic tissues (Fig. 6G, 6H), further supporting our discovery that upregulated USP22 in breast cancer stem cells though FoxM1-mediated *ITGB1* gene transcription for promoting breast cancer lung metastasis. To support this notion, the protein expression levels of USP22, FoxM1 and integrin b1 are strongly correlated in human breast cancers (Fig. 6I & s6E). Collectively, our study identified USP22 as a FoxM1-specific deubiquitinase which promotes FoxM1 transcriptional activation for *ITGB1* expression, which consequently promotes breast cancer stem cell self-renewal and drives breast cancer metastasis to distal organs including lung (Fig. 6J).

## Discussion

Our study defines a previously unknown USP22-FoxM1-integrin b1 pathway critically important for both mouse and human breast cancer stem cell self-renewal. This conclusion is documented by the following discoveries: First, USP22 is further upregulated in BCSCs and breast cancer and targeted USP22 deletion dramatically impaired BCSC self-renewal and tumorigenicity; Second, USP22 controls breast cancer cell stemness through integrin b1 upregulation; Third, USP22 functions as a bona fide deubiquitinase of the *ITGB1* transcription FoxM1 and promotes BCSC self-renewal through FoxM1-mediated integrin b1 expression. Fourth, pharmacological USP22 inhibition impairs BCSC self-renewal and protects mice from breast cancer lung metastasis-induced mortality; Last but not least, USP22 and *ITGB1* are positively correlated in more than 90% of human cancer types, and USP22, integrin b1 and FoxM1 are increased and positively correlated in breast cancers.

Integrin signals play critical roles in supporting the function of both normal adult stem cells and their neoplastic derivatives ^32^. While integrin mutations are rarely identified, most of, if not all integrin family members are often upregulated in cancer cell stem cells and this upregulation often promotes CSC self-renewal, cancer initiation and metastasis ^19, 33, 34^. Several tumor initiating and/or promoting pathways including epidermal growth factor (EGF) and vascular endothelial growth factor-mediated signaling pathways activate the RAS-MAP kinase cascade for *ITGB1* transcription through downstream AP-1 family transcription factors. On the other hand, the tumoral immune suppressive cytokine TGF-b promotes b1 integrin expression through canonical SMAD family transcription factor activation. In addition, the fork head family transcription factors, both FoxO3 and FoxM1 have been identified to promote cancer invasion through promotes b1 ^27, 35^. Our studies here define the USP22-FoxM1-integrin b1 axis as critical regulatory node in control of breast cancer stem cell self-renewal, tumor initiation and metastasis. In addition to integrin b1, USP22 appears to promote the transcription of several additional integrin family members. However, FoxM1 reconstitution only rescued integrin b1 expression, implying that USP22 regulates integrin family members via district molecular mechanism. FoxM1 has been also known as a crucial transcription factor for the maintenance of a variety of human CSCs and its expression is associated with a worse clinical prognosis ^36, 37, 38^. Therefore, this study links three important cancer stem cell genes teaming together to maintain an optimal breast cancer stem cell pool. Importantly, our unbiased analysis of existing public database revealed a statistically significant positive correlation between USP22 and *ITGB1* in 37 total human cancer types, suggesting that the USP22-FoxM1-integrin b1 axis is a common mechanism in CSC self-renewal.

We also noticed that, while integrin b1 expression is full restored in USP22-null mouse and human breast cancer cells, FoxM1 expression only achieved a partial rescue in their *in vitro* tumor sphere formation and *in vivo* lung metastasis, indicating that USP22 exerts it cancer stem cell gene function in part through an integrin b1-indepent manner. Indeed, it has been shown that USP22 promotes hypoxia-induced hepatocellular carcinoma stemness through a HIF-1a/USP22 positive feedback loop upon TP53 inactivation ^39^. On the other hand, USP22 regulates embryonic stem cell differentiation via transcriptional repression of sex-determining region Y-box 2 (SOX2) ^40^. Therefore, USP22 appears to play a diverse role in regulating cell stemness in both physiological and pathological contexts.

Our study provides a strong rationale for targeting the USP22-FoxM1-integrin β1 pathway in anticancer therapy. In fact, pharmacological USP22 inhibition dramatically reduced the frequency of breast cancer stem cells and attenuated both mouse and human invasive breast cancer lung metastasis. In addition to its cancer cell-intrinsic roles, USP22 has been recently discovered to suppress tumor immunosurveillance through potentiating Foxp3 + regulatory functions ^31,41^ as well as upregulating the expression of checkpoint receptors PD-L1 and CD73 ^42, 43^. Therefore, USP22 targeting presumably achieves both chemo- and immune-therapeutic efficacy. On the other hand, either specific antibody or peptide inhibitors of integrin family members has been tested for antitumor therapy and several clinical trials are still on going. The anti-a5b1 integrin antibody volociximab was shown to inhibit angiogenesis and suppress tumor growth and metastasis in mice and show some antitumor efficacy in treatment of the advanced non-small-cell lung cancer and in pancreatic cancer ^44, 45^. However, directly targeting integrin b1 has only achieved very limited success as integrin b1 is highly expressed in a variety of normal cells and required for critical biological functions including normal mammary stem cells maintenance ^46^. Importantly, our discovery that USP22-FoxM1-integrin b1 pathway is critical for breast cancer self-renewal indicates that simultaneous USP22 and integrin b1 targeting may achieve a synergistic efficacy in combating human cancers, which leads to reduced therapeutic doses and side effects from both sides.

## Materials And Methods

### Cell culture

Human HEK-293T cells were cultured in DMEM medium plus 10% FBS (Thermo Fisher Scientific,0437028) and 1% penicillin and streptomycin. 4T1 cells were maintained in RPMI medium supplemented with 10% FBS and 1% penicillin and streptomycin. TN1 cells were cultured in HuMEC-ready medium (Life Technologies) supplemented with 5% FBS and 0.5% P/S in collagen type I (BD Biosciences) coated plates.

### Molecular cloning and plasmid

Mice and human FoxM1 overexpressed plasmid were purchased from addgene and subclone into pCMV plasmids. Human or mouse USP22 single guide RNA sequence was ligated into lentiCRISPR v2 plasmid separately. Indicated cells were transiently transfected using TurboFect (Thermo Fisher). 48 hours after transfection, cells were selected using puromycin for 14 days. The efficacy of USP22 deletion was validated by western blotting. The sequences of each guide RNA used in this study was shown Supplementary Table 3.

### Tumor sphere formation assay

A total of 3×10^4^ 4T1 or TN1 cells expressing with or without USP22 sgRNA were plated into ultralow-attachment 6-well plates (Corning, Cat#3471), and maintained in EpiCult-B Basal Medium (Human) (Stem Cell Technologies, BC, Canada) and EpiCult-B Proliferation Supplement (Human) (Stem Cell Technologies, BC, Canada), and supplemented with 2 U/mL heparin and 0.5 mg/mL hydrocortisone (Sigma H0135). After 10 days culture, the spheres were pictured, and the number of spheres in each group were counted.

### Colony formation assay

A total of 300 indicated cells were seeded into 35 mm dishes with triplicates, and maintained in culture for two weeks. The culture medium was changed every 3 days. When colonies grew to visible size, the colonies were then washed twice with phosphate buffered saline and fixed with 4% formaldehyde for 30 min at room temperature and stained for 1 h with 0.1% crystal violet. After staining, the plates were gently washed with distilled water and air-dried. The exact colony number of colonies was then quantified by ImageJ software.

### *In vitro* extremely limiting dilution assay

Indicated cells were dissociated into single cell suspensions and seeded into 96-well plates at density of 5, 10, 15, 20 cells per well using previous mentioned tumor sphere formation medium. Cells were incubated at 37 °C for 10 to 14 days. At the time of quantification, each well was exactly counted for formed tumor spheres. Stem cell frequency was calculated using extreme limiting dilution analysis online tool ^47^ (http://bioinf.wehi.edu.au/software/elda/).

### Real-time PCR

Total RNA was extracted from indicated cells using Trizol. The cDNA was synthesized using a Quantifect Reverse Transcription Kit. qRT-PCR was performed using SYBR Premix Ex Taq, primers, H_2_O, and cDNA (final reaction volume, 20 mL). The sequences of the primers used in this study were shown in Supplementary Table 3.

### Flow cytometry analysis and cell sorting

For CD24^−^/CD44^+^ BCSCs sorting, 4T1 and TN1 cells were washed with PBS, dissociated using accutase, counted and incubate with primary antibody against CD44 and CD24 on ice for 60 minutes. FacsAria (BD) cell sorter equipment was used to isolate CD24^−^/CD44^+^ and CD24^+^/CD44^−^ cells, respectively. For the 4T1 GFP-USP22 fusion knock-in cells, cells were dissociated using accutase. Cells were then sorted using FacsAria (BD) cell sorter equipment based on fluorescence intensity. For integrin family expression evaluation, indicated cells were dissociated using accutase and followed by staining with indicated antibodies on ice for 60 minutes. Cells were run on the BD-LSR Fortessa X-20 (BD Biosciences) instrument and flow analyses were done using FlowJo software. The detailed information of antibodies used in flow cytometry were shown in Supplementary Table 3.

### Immunoblot

Indicated cells in this study were lysed with RIPA buffer supplemented with protease inhibitors. The same quality of protein was subjected to SDS-PAGE gel electrophoresis, transferred onto polyvinylidene fluoride membranes, and blocked with 5% skimmed milk for 30 min at room temperature. The membranes were then incubated with primary antibodies. The detailed information of antibodies used in this study were shown in Supplementary Table 3.

### Co-immunoprecipitation

TN1 or 4T1 cells were harvested and lysed with RIPA buffer containing protease inhibitors. Cell lysates were precleared using protein A/G beads (10294276, GE healthcare) for 1 h incubation with gentle shake at 4 °C, and precleared protein A/G beads were removed and followed by adding primary antibody for overnight incubation with gentle shake at 4 °C, and new protein A/G beads were subsequently added for another 2 h incubation, then beads were collected following washing with ice-cold PBS for 4 times. Finally, the bound protein was eluted by boiling for 5 min and subjected to SDS-PAGE.

### Immunohistochemistry

Immunohistochemical (IHC) staining was performed following the standard protocol as reported ^48, 49^. Briefly, tissue specimens were subjected to deparaffinized in xylene, rehydrated through graded ethanol solutions, antigen retrieval and immersed in a 0.3% hydrogen peroxide solution. After carefully washing three times with phosphate-buffered saline (PBS), and nonspecific antigen was then blocked by incubation with 5% bovine serum albumin for 30 min at room temperature. The tissue slides were subsequently incubated with primary antibodies overnight at 4 °C. Horseradish peroxidase (HRP) conjugated secondary antibody was used to incubate the slides before DAB detection. For the IHC results analysis, the percentage score was assigned as follows: 1 indicated that 0–25% of the tumor cells showed positive signaling, 2 indicated 26–50% of cells were stained, 3 indicated 51–75% stained, and 4 indicated 76–100% stained. We scored the staining intensity as 0 for negative, 1 for weak, 2 for moderate, and 3 for strong. The total score was obtained by multiplying the percentage score by the stain intensity score. The detailed information of antibodies used in IHC were shown in Supplementary Table 3.

### Animal studies

All animal experiments were approved by the respective Institutional Animal Care and Use Committee at Northwestern University. All mice were maintained in a specific pathogen-free facility. BALB/c, and NSG mice at the age of 6–8 weeks were all purchased from Jackson laboratory. For the metastatic mice model, BALB/c mice were intravenously administrated with 5×10^4^ 4T1 USP22 ablation or control cells. 20 days later, all the mice were sacrificed and analyzed the metastatic nodules. For the survival analysis of mice, BALB/c mice were intravenously administrated with 5×10^4^ 4T1 wildtype cells, mice were euthanized until exhibiting signs of significantly declining their quality of life (e.g., ataxia, lethargy, seizures, inability to feed) and the survival of mice were recorded. For the S02 treatment, BALB/c mice were intravenously administrated with 5×10^4^ 4T1 cells. 24 hours later, mice were randomized into treatment groups and treated with S02 (10 mg/kg), or vehicle control by intraperitoneal injection six times (once every day). Mice were sacrificed 3 weeks later after 4T1 cells administration and the lung of mice were taken out to analyze tumor nodules. For the orthotopic xenograft model, 5×10^4^ TN1 cells were orthotopically injected into the mammary fat pad of NSG mice, 2 weeks later, mice were randomized into treatment groups and treated with S02 (20 mg/kg), or vehicle control group by intraperitoneal injection six times (twice every day).

### Statistical analysis

Data are represented as the mean ± SD, and error bars indicate SD. *P* values were calculated by either unpaired or paired two-tailed Student’s t test, *P< 0.05, **P< 0.01, and ***P< 0.001. All analyses were performed using GraphPad Prism software (GraphPad Software, Inc.).

## Data Availability

The data are available to academic researchers from corresponding author upon reasonable request.
